# Genome-wide identification, organization and phylogenetic analysis of Dicer-like, Argonaute and RNA-dependent RNA Polymerase gene families and their expression analysis during reproductive development and stress in rice

**DOI:** 10.1186/1471-2164-9-451

**Published:** 2008-10-01

**Authors:** Meenu Kapoor, Rita Arora, Tenisha Lama, Aashima Nijhawan, Jitendra P Khurana, Akhilesh K Tyagi, Sanjay Kapoor

**Affiliations:** 1 University School of Biotechnology, Guru Gobind Singh Indraprastha University, Kashmere Gate, Delhi-110006, India; 2 Interdisciplinary Centre for Plant Genomics and Department of Plant Molecular Biology, University of Delhi South Campus, Benito Juarez Road, New Delhi-110021, India

## Abstract

**Background:**

Important developmental processes in both plants and animals are partly regulated by genes whose expression is modulated at the post-transcriptional level by processes such as RNA interference (RNAi). Dicers, Argonautes and RNA-dependent RNA polymerases (RDR) form the core components that facilitate gene silencing and have been implicated in the initiation and maintenance of the trigger RNA molecules, central to process of RNAi. Investigations in eukaryotes have revealed that these proteins are encoded by variable number of genes with plants showing relatively higher number in each gene family. To date, no systematic expression profiling of these genes in any of the organisms has been reported.

**Results:**

In this study, we provide a complete analysis of rice Dicer-like, Argonaute and RDR gene families including gene structure, genomic localization and phylogenetic relatedness among gene family members. We also present microarray-based expression profiling of these genes during 14 stages of reproductive and 5 stages of vegetative development and in response to cold, salt and dehydration stress. We have identified 8 Dicer-like (*OsDCLs*), 19 Argonaute (*OsAGOs*) and 5 RNA-dependent RNA polymerase (*OsRDRs*) genes in rice. Based on phylogeny, each of these genes families have been categorized into four subgroups. Although most of the genes express both in vegetative and reproductive organs, 2 *OsDCLs*, 14 *OsAGOs *and 3 *OsRDRs *were found to express specifically/preferentially during stages of reproductive development. Of these, 2 *OsAGOs *exhibited preferential up-regulation in seeds. One of the Argonautes (*OsAGO2*) also showed specific up-regulation in response to cold, salt and dehydration stress.

**Conclusion:**

This investigation has identified 23 rice genes belonging to DCL, Argonaute and RDR gene families that could potentially be involved in reproductive development-specific gene regulatory mechanisms. These data provide an insight into probable domains of activity of these genes and a basis for further, more detailed investigations aimed at understanding the contribution of individual components of RNA silencing machinery during reproductive phase of plant development.

## Background

In plants, small RNAs have been widely implicated in varied developmental events and as guide RNAs in many gene silencing pathways [1-6]. These RNAs are usually generated by the activities of Dicers, Argonautes and RNA-dependent RNA polymerases (RDRs), which are also sometimes referred to as the core proteins mediating RNA interference. These proteins are involved in the initiation and maintenance of the trigger RNA that is central to this mode of gene regulation. Briefly, initiation of gene silencing involves generation of double stranded RNA (dsRNA) by several mechanisms, for example, bidirectional transcription of DNA, self-complementary RNA foldbacks or RNA-dependent transcription of aberrantly synthesized mRNAs [7]. The complementary dsRNAs are then processed by the RNaseIII-type activities of Dicers into small RNAs, ~19–31 nucleotides in length (siRNA or miRNA). Generally, one strand of these RNAs then associates with the silencing effector complexes through the Argonaute proteins. This confers sequence-specific guide functions to these complexes that find target RNAs with sequences complementary to the small RNAs. Silencing/repression of the target genes then occurs by either blocking translation or cleavage of the target mRNA. These small RNAs could also mediate transcriptional gene silencing by recruitment of histone and/or DNA methyltransferases to regulatory sequences of the target genes [8,9].

Dicers and Argonautes are multidomain ribonucleases. Dicers are characterized by the presence of six types of domains, viz., DExD-helicase, helicase-C, Duf283, PAZ, RNaseIII and double stranded RNA-binding (dsRB) domain [10]. All Argonaute proteins share the domain structure that comprises of an N terminal, PAZ, Mid and a C-terminal PIWI domain. The PAZ domain (~100 a.a) facilitates binding of 3' end of siRNA, while, the PIWI domain binds the 5' end of siRNA and to the target RNA. This domain has marked similarity with RNaseH family of ribonucleases and it possesses the catalytic amino acid residues required for endonucleolytic cleavage of the target RNA [11]. At least three subfamilies of Argonaute proteins have been identified in eukaryotes [12]. These include the AGO subfamily that is present in plants, animals and yeasts; the Piwi subfamily that has been found only in animals and the worm-specific Argonaute (WAGO) subfamily that are present in *C. elegans *[13,14,6]. Members of both AGO and Piwi subfamilies possess the characteristic DDH metal binding signature residues in their Piwi domains, while most of the WAGO proteins lack them. The Piwi proteins are expressed specifically in the germline cells and are known to interact with a subset of small RNA called Piwi-interacting RNA that are longer (26–31 nt) than siRNA and miRNA (21–24 nt) [13]. This clade of Argonaute proteins have not been identified in any plant species. Recently, however, a novel kind of Argonaute, *OsMEL1 *has been described in rice that is involved specifically in male meiosis [15]. RNA-dependent RNA polymerase was first isolated from tomato [16]. These proteins are required for initiation and amplification of silencing signal and they possess a conserved sequence motif that resembles the catalytic β' subunit of DNA-dependent RNA polymerases [17].

Multiple copies of Dicers, Argonautes and RDR genes are known to exist in both plants and animals. With the exception of fission yeast (*Schizosaccharomyces pombe*) that codes for only one copy each of the Dicer, the Argonaute and the RDR, these proteins are encoded by multigene families in insects, nematodes, mammals and plants [18]. In *Arabidopsis*, 4 Dicer-like (DCL), 10 Argonautes and 6 RDR genes have been identified. Functions of few of these genes have also been elucidated. *Arabidopsis Dicer-like 1 *(*DCL1*), but not *DCL2*, *3 *and *4*, has been implicated in biogenesis of miRNA [19]. *ARGONAUTE1 (AGO1) *regulates floral meristem and organ identity by affecting expression of *LEAFY*, *APETALA1 *and *AGAMOUS *genes. It also affects expression of the polycomb group protein, *CURLYLEAF *(*CLF*) that maintains repression of both *KNOX *and *AGAMOUS *in vegetative organs [20]. In *Drosophila*, *DICER-1 (Dcr-1) *has been found to be essential for miRNA formation and it functions redundantly with *Dcr-2 *downstream of siRNA production. *Dcr-2*, on the other hand, has also been implicated in siRNA formation [21,22]. In *Neurospora crassa*, distinct RDR and Argonaute proteins are required for meiotic silencing of unpaired DNA (in nucleus) and RNAi (in cytoplasm; [23]). In *Caenorhabditis elegans*, the 22 nucleotide endogenous miRNA encoded by *lin-4 *and *let-7 *genes are processed by *Dcr-1 *and these small RNAs regulate *lin-14 *and *lin-41 *linked timing of developmental stages in the nematode life cycle [24].

In rice, components of the RNAi machinery are known to be involved in maintenance of undifferentiated cells in shoot apical meristem (SAM), initiation of lateral organ primodia from SAM and floral meristems and formation of male and female germ cells [15,25,26]. The present investigation has been carried out with the aim to obtain comprehensive expression overview of all the members of rice Dicer-like, Argonaute and RDR gene families to gain insight into the domains of activity of these genes and to provide a firm foundation for further, more detailed investigations aimed at understanding the contribution of individual components of RNA silencing machinery in regulating gene expression during reproductive development of plants. For this, an in-house generated (previously described; [27]) rice microarray data set comprising of 17 stages of vegetative and reproductive development, along with 5 newly added tissues/developmental stages corresponding to Y-leaf, SAM and three very early stages of panicle initiation, was utilized. Where possible, the expression profiles of rice genes have been compared with those in *Arabidopsis *at similar stages of flower and seed development to identify genes with similar expression profiles in dicots.

## Results

### Identification and structural organization of rice Dicer-like, Argonaute and RDR genes

Name search using the keywords, Dicer, PAZ, PIWI, Argonaute and RNA-dependent RNA polymerase and HMM analysis resulted in the identification of 8 genes encoding Dicer-like (*OsDCL*) proteins, 19 for Argonautes (*OsAGO*) and 5 genes for RDR (*OsRDR*) in the rice genome (TIGR rice pseudomolecule release 5). Besides confirming the previously identified gene members, this exercise revealed two additional *OsDCL *and one Argonaute encoding loci [10]. The newly identified Dicer-like loci are LOC_Os05g18850 (*OsDCL1c*) and LOC_Os06g25250 (*OsDCL1b*) with coding potential of 318 and 300 amino acid polypeptides, respectively (Table 1). Protein evidence for only LOC_Os06g25250 was found in the Swiss-Prot/TrEMBL database (accession number: Q69KJ0). Search for the conserved domains by Simple Modular Architecture Research Tool (SMART) analysis and in NCBI databases revealed the presence of only RNaseIII and dsRB domains in these proteins, while in most other OsDCLs, DExD, Helicase-C, Duf283, PAZ, RNaseIII and dsRB domains, characteristic of plant DCL proteins, were present. OsDCL2b, however, lacked the Duf283 domain and both OsDCL2a and 2b lacked one of the dsRB domains, as reported previously [10]. Maximum number of introns, 26 in number, was found in *OsDCL3a *that codes for a 1598 amino acid polypeptide.

**Table 1:** Structural characteristics of Dicer-like, Argonaute and RNA dependent RNA Polymerase genes identified in rice

**Serial No.**	**Gene Name**	**Accession Numbers of Gene Models**	**TIGR Locus ID**	**Coordinates (5'-3')**	**ORF Length (bp)**	**Protein**			**No. of Introns**
							
		**TIGR (Release 5)**				**Length (a.a.)**	**Mol. Wt. (Da)**	**pI**	
**DICER-like**									
1	*OsDCL1a*	12003.m05835	LOC_Os03g02970	1183184 – 1174351	5655	1884	210202.11	6.64	18
2	*OsDCL2a*	12003.m08967	LOC_Os03g38740	21469900 – 21455783	4236	1411	158493.38	6.86	19
3	*OsDCL2b*	12009.m04723 12009.m50214 12009.m50215	LOC_Os09g14610	8649185 – 8646060	1080	359	39984.82	7.27	2
4	*OsDCL3a*	12001.m12915	LOC_Os01g68120	39932566 – 39922935	4797	1598	178239.94	6.65	26
5	*OsDCL3b*	12010.m06268	LOC_Os10g34430	18031152 – 18042777	4719	1572	177717.08	6.7	25
6	*SHO1*	12004.m09269 12004.m101860 12004.m78968 12004.m78969	LOC_Os04g43050	25262283 – 25255247	3180	1059	119863.82	7.55	25
7	*OsDCL1c*	12005.m06289	LOC_Os05g18850	10924931 – 10927291	957	318	34954.29	8.7	2
8	*OsDCL1b*	12006.m07173	LOC_Os06g25250	14769389 – 14772506	903	300	33335.89	5	2
**ARGONAUTES**									
1	*OsAGO1a*	12002.m09547 12002.m100310	LOC_Os02g45070	27335674 – 27324616	3252	1083	**120463**	**9.43**	22
2	*OsAGO1b*	12004.m09728 12004.m101624	LOC_Os04g47870	28214275 – 28207729	3309	1102	121632.35	9.86	22
3	*OsAGO1c*	12002.m10870	LOC_Os02g58490	35762233 – 35755714	3039	1012	**113158**	**9.53**	22
4	*OsAGO1d*	12006.m091803 12006.m09654	LOC_Os06g51310	31069900 – 31076078	3120	1039	115923.39	9.27	22
5	*OsAGO2*	12004.m10147	LOC_Os04g52540	31020218 – 31024093	3105	1038	111444.34	9.7	2
6	*OsAGO3*	12004.m10148	LOC_Os04g52550	31025382 – 31030370	3333	1110	122752.46	9.45	4
7	*OsAGO4a*	12001.m08283 12001.m150415 12001.m150416 12001.m150417	LOC_Os01g16870	9633946 – 9628308	2718	905	100638.84	9.32	21
8	*OsAGO4b*	12004.m05971 12004.m35113	LOC_Os04g06770	3547911 – 3542867	2739	912	101739.93	9.2	21
9	*OsAGO14*	12007.m05358	LOC_Os07g09020	4710731 – 4701446	3162	1053	113909.58	10	21
10	*OsMEL1*	12003.m10765	LOC_Os03g58600	33319166 – 33310479	3180	1059	117034.04	9.58	21
11	*OsAGO13*	12003.m10673	LOC_Os03g57560	32753781 – 32760315	3186	1061	121023.51	9.46	21
12	*OsAGO16*	12007.m06064	LOC_Os07g16224	9466401 – 9459149	1095	364	40509.71	8.07	10
13	*SHL4*	12003.m08604	LOC_Os03g33650	19198976 – 19203042	3168	1055	118110.77	9.76	2
14	*OsPNH1*	12006.m08501	LOC_Os06g39640	23539785 – 23545931	2925	974	107717.15	9.64	21
15	*OsAGO17*	12002.m06078	LOC_Os02g07310	3754861 – 3748703	2634	877	**98923**	**8.64**	21
16	*OsAGO12*	12003.m09795	LOC_Os03g47820	27110631 – 27102684	2934	977	107073.06	9.83	21
17	*OsAGO11*	12003.m09796	LOC_Os03g47830	27127103 – 27114562	2688	895	99845.06	8.94	20
18	*OsAGO18*	12007.m07156	LOC_Os07g28850	16891936 – 16898240	3270	1089	118034.34	9.57	20
19	*OsAGO15*	12001.m08281	LOC_Os01g16850	9622035-9610432	6711	2237	246763.92	8.51	19
**RNA-DEPENDENT RNA POLYMERASES**									
1	*SHL2*	12001.m09741	LOC_Os01g34350	19272378 – 19267489	3657	1219	136533.79	7.11	1
2	*OsRDR2*	12004.m08905	LOC_Os04g39160	23065706 – 23070916	3411	1137	126924.77	7.56	3
3	*OsRDR4*	12001.m07636 12001.m97449 12001.m97489	LOC_Os01g10140	5321158 – 5304556	3621	1207	135030.19	7.1	18
4	*OsRDR1*	12002.m10067	LOC_Os02g50330	30727018 – 30730508	2223	741	**84265**	**6.88**	2
5	*OsRDR3*	12001.m07635	LOC_Os01g10130	5289905 – 5285242	1311	437	50679.98	6.57	6

Values in Bold: Molecular weight and pI calculated by Gene Runner as it was not available in TIGR bp, base pairs; a.a., amino acid; Da, Dalton

**Figure 1:**
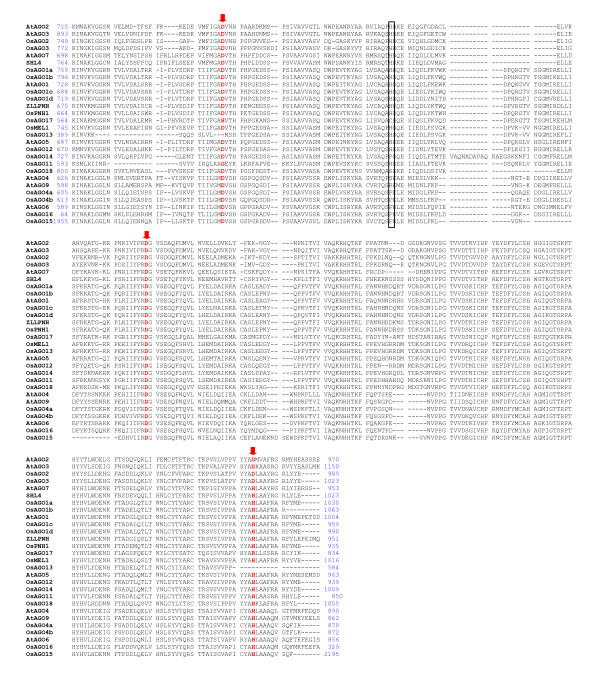
**Amino acid alignment of Piwi domains of rice and *Arabidopsis *AGO proteins**. The protein sequences were aligned using clustalX (1.83). The conserved Asp, Asp and His (DDH) triad residues are marked with downward arrows, while the His (H) corresponding to H798 of *Arabidopsis *AGO1 have been boxed. Amino acid positions corresponding to the beginning and end of the Piwi domains in each protein are mentioned.

Of the 19 Argonautes identified in rice in the present study, 17 and 18 genes respectively, were reported recently [15,28]. The additional *OsAGO *(named *OsAGO4b*) identified in this study, corresponds to the locus LOC_Os04g06770 and is characterized by the presence PAZ and PIWI domains in the putative polypeptide sequence. All *OsAGOs *code for ~100 kDa basic proteins with pI ranging from 8.07–10. The OsAGOs are characterized by the presence of PAZ domain (~100 amino acids) towards the amino terminus and PIWI domain (~400 amino acids) at the carboxyl end. As an exception, *OsAGO16 *was found to code for only the PIWI domain (Table 1). *OsAGO15 *(LOC_Os01g16850) is characterized by the longest open reading frame of 6711 bp with a coding potential for a 2237 amino acid polypeptide. This protein has been annotated as a retrotransposon protein in TIGR release 5. However, it possesses the Argonaute-specific PAZ and PIWI domains besides the DUF, intergrase core domain (rve) and the reverse transcriptase (RVT_2) domain, characteristic of mobile elements. This gene has also been included as member of the rice Argonaute gene family in previous studies [15,28]. Structural studies in other organisms have shown that the PIWI domain of Argonaute proteins folds similar to RNaseH proteins. Consistent with this observation, some Argonaute proteins in both plants and animals are known to cleave the target RNAs that have sequence complementary to the small RNAs [11,18]. These catalytic proteins are known to possess three conserved metal chelating residues in the PIWI domain i.e. aspartate, aspartate and histidine (DDH), that function as the catalytic triad. In *Arabidopsis *AGO1, a conserved histidine at position 798 (H798) was also observed to be critical for the endonuclease activity of AGO1 *in vitro *[18]. To interrogate which of the OsAGO possessed the conserved catalytic residues and could potentially act as the slicer component of silencing effector complexes, we aligned the PIWI domains of all the OsAGOs using CLUSTALX (Figure 1). Eight proteins, namely OsAGO1a, OsAGO1b, OsAGO10a, OsAGO10b, OsMEL1, OsAGO7, OsPNH1 and OsAGO12, were found to have the conserved DDH/H798 residues. In three OsAGOs, the first aspartate was either missing or was replaced by a glycine or histidine. The other six possessed the conserved DDH triad but the histidine at 798^th ^position in *AGO1 *was either replaced by serine or a proline (Table 2). At gene structure level, the number of introns in *OsAGO *varied from 2 in *OsAGO2 *and *SHL4 *to 22 in *OsAGO1a, OsAGO1b, OsAGO1c *and *OsAGO1d *(Table 1).

**Table 2:** Rice and *Arabidopsis *Argonaute proteins with missing catalytic residue(s) in PIWI domains

**S. No.**	**Rice**		***Arabidopsis***	
	
	**Argonaute**	Motifs*	**Argonaute**	Motifs*
1	OsAGO2	DDD/H	AGO2	DDD/H
2	OsAGO3	DDD/H	AGO3	DDD/H
3	OsAGO13	-D-/H	AGO4	DDH/S
4	OsAGO11	GDH/H	AGO9	DDH/R
5	OsAGO17	HDR/C	AGO6	DDH/P
6	OsAGO14	DDH/P		
7	OsAGO18	DDH/S		
8	OsAGO4a	DDH/P		
9	OsAGO4b	DDH/P		
10	OsAGO15	DDH/P		
11	OsAGO16	DDH/P		

*Motifs correspond to conserved D760, D845, H986/H798 of *Arabidopsis *AGO1

All the 5 RDR genes present in the rice genome were found to encode proteins that share a common sequence motif corresponding to the catalytic β' subunit of DNA-dependent RNA polymerases [17]. The length of the open reading frames of *OsRDR*s varied from 1311 bp for *OsRDR3 *to 3657 bp for *SHL2*, with the coding potential of 437 and 1219 amino acids, respectively. Interestingly, the genomic sequence of *SHL2 *that encodes the longest open reading frame is interrupted by a single intron, while *OsRDR4 *that encodes the second longest open reading frame of 3621 bp has 18 intervening sequences as annotated in TIGR (release 5; Table 1).

### Phylogeny and chromosomal localization

**Figure 2:**
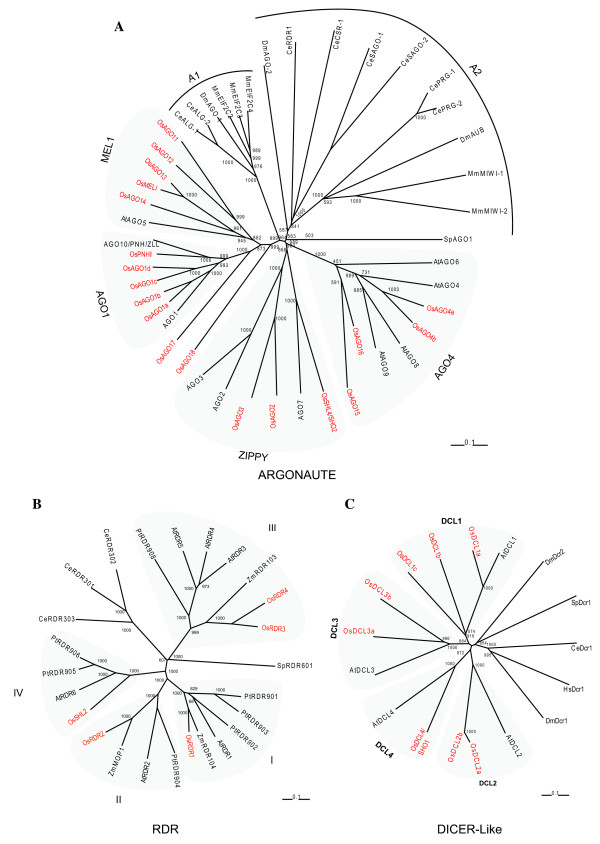
**Phylogenetic analysis of Dicer-like, Argonaute and RDR genes of rice with other organisms**. **A. Argonautes**. Unrooted, neighbor joining tree was constructed by alignment of total protein sequences from rice, *Arabidopsis, C. elegans*, yeast, Drosophila and humans. Four subgroups are marked, AGO1, MEL1, ZIPPY and AGO4 as described by Nonomura et al., (2007, [15]). Plant-specific clades are shaded and rice Argonautes have been highlighted in red in each group. Accession numbers of sequences are as follows: CeALG-1 (NP510322), CeALG-2 (NP493837), CePRG-1(CAA98113), CePRG-2 (AAB37734), CeRDE-1 (AAF06159), DmAGO1(NP523734), DmAGO2 (AAF49619), DmAUB (CAA64320), MmMIWI1(NP067286), MmMIWI2 (AY135692), MmEIF2C2 (NP694818), MmEIF2C3 (NP700451), MmEIF2C4 (NP694817), SpAGO1 (CAA19275), CeCSR-1 (NP001040938), CeSAGO-1 (NP504610), and CeSAGO-2 (NP490758), AtAGO10 (AAD40098), AtAGO1 (AAC18440), AtAGO7 (AAQ92355) and AtAGO4 (NP_565633), AtAGO2 (Locus:2197545), AtAGO3 (Locus:2197550), AtAGO6 (Locus:2059370), AtAGO8 (Locus:2147072) and AtAGO9 (Locus:2179008). **B. RNA-dependent RNA polymerase**. Analysis of evolutionary relatedness between rice, *Arabidopsis*, Maize, Populus, yeast and *C. elegans *RDR genes using full length RDR protein sequences. The protein sequences of all RDRs except rice were obtained from the Chromatin database, ChromDB . Plant-specifc clades have been shaded. The *Arabidopsis *proteins are AtRDR1(At1g14790), AtRDR2 (At4g11130), AtRDR3 (At2g19910), AtRDR4 (At2g19920), AtRDR5(At2g19930), and AtRDR6 (At3g49500). PtRDR901 through PtRDR906 and PtRDR908 are *Populus trichocarpa *proteins, ZmRDR103, ZmRDR104 and ZmMOP1 are *Zea mays *RDR proteins, SpRDR601 is from *Schizosaccharomyces pombe *and CeRDR301-CeRDR303 are from *Caenorhabditis elegans*. **C**. **Dicer-like proteins**. Neighbour-joining tree was constructed after alignment of total protein sequences of rice and *Arabidopsis*. Protein sequences from Drosophila, humans, yeast and *C. elegans *were included as outgroups. Plant-specific clades have been shaded. Protein sequences were downloaded from National Center for Biotechnology Information (NCBI). Accession numbers and abbreviations are as follows: HsDcr1, *Homo sapiens *Dicer-1 (NP_085124); DmDcr1, *Drosophila melanogaster*, Dicer-1(NP_524453); DmDcr2, *Drosophila melanogaster *Dicer-2 (NP_523778) CeDcr1, *Caenorhabditis elegans*, Dcr-1(AAA28101); SpDcr1, *Schizosaccaromyces pombe*, Dcr-1 (Q09884); AtDCL1, *Arabidopsis thaliana *DCL1, (NP_171612); AtDCL2, *Arabidopsis thaliana *DCL2, (NP_566199), AtDCL3, *Arabidopsis thaliana *DCL3, (NP_189978); AtDCL4, *Arabidopsis thaliana *DCL4, (NP_197532). Rice Dicer-like proteins are shown as OsDCL1a to OsSHO1/DCL4. Scale bar in each panel represents 0.1 amino acid substitution per site.

To determine evolutionary relatedness of rice Argonautes, RDRs and DCLs with those from mammals, *Drosophila*, *Caenorhabditis elegans*, *Schizosaccharomyces pombe *and *Arabidopsis*, total protein sequences from these organisms were used to construct an unrooted neighbour-joining phylogenetic tree (Figure 2). Rice and *Arabidopsis AGO *genes clustered into four subgroups, *MEL1, AGO1, AGO4 *and *ZIPPY *(Figure 2A), similar to those described by Nonomura *et al*. (2007; [15]). In *AGO1 *subgroup, four rice genes grouped with single *Arabidopsis *gene *AGO1 *that is closely related to *AGO10/PNH*. These genes have been designated as *OsAGO1a*, *OsAGO1b*, *OsAGO1c *and *OsAGO1d *on the basis of high sequence similarity to *AGO1 *and similar gene expression profiles to the *Arabidopsis *gene (described elsewhere). The MEL1 group contains five rice genes including *OsMEL1 *and the only *Arabidopsis *gene *AGO5*. Comparison of full-length OsAGO proteins of this clade revealed that these proteins shared 25 – 60% overall identity but their PIWI domains had 75 – 94% similar residues. Since only *AGO5 *and *OsMEL1 *exhibit similar expression profiles, other genes in this clade have been named *OsAGO11-14*. In the AGO4 subgroup, two highly similar rice members, LOC_Os04g06770 and LOC_Os01g16870 have been named *OsAGO4a *and *OsAGO4b *as they share greater similarity with *AGO4 *(more than 65% at amino acid level) in comparison to *AGO9*, *AGO8 *and *AGO6*. The animal Argonautes, on the other hand, are grouped into two distinct clades A1 and A2. The phylogenetic relationship of these groups suggests that the plant AGO1 and MEL1 clades had a common lineage with A1, while ZIPPY and AGO4 clades may have diverged from an ancestral lineage that gave rise to A2 clade in animals.

Phylogenetic analysis of rice, *Arabidopsis*, *Zea mays*, *Populus trichocarpa*, *C. elegans *and *Schizosaccharomyces pombe *RDR genes revealed that dicot and monocot RDR genes cluster into four clades, I, II, III and IV (Figure 2B). Members in each clade show monophyletic pattern of origin. The rice RDR genes have been named as *OsRDR1 *to *4 *on the basis of sequence similarity with the corresponding proteins in *Arabidopsis*. LOC_Os01g10130 was named *OsRDR3 *and LOC_Os01g10140 has been named *OsRDR4 *on the basis of phylogeny and the results of homology scores of the rice genes with the corresponding *Arabidopsis RDR3 *and *RDR4 *genes in BLAST searches (data not shown). The RDR genes of *C. elegans *also originated from a common ancestral gene that diverged from the clades that gave rise to plant and the single yeast gene.

Plant *DCL *genes form a monophyletic group with *OsDCL *genes showing high sequence conservation with their counterparts in *Arabidopsis *(Figure 2C). Dicers from *Drosophila (Dcr-1)*, nematode and humans form one group that is distinct from *Drosophila Dicer2 *and the only Dicer from *S. pombe *due to absence of the conserved PAZ domain in these proteins (Figure 2C). The newly identified *OsDCL *loci closely grouped with the highly similar *AtDCL1 *and *OsDCL1*. Similar associations were observed when only dsRBa domains of rice and *Arabidopsis *proteins were used to construct a phylogenetic tree (data not shown). Therefore, we have renamed *OsDCL1 *as *OsDCL1a*, and named the two new loci as *OsDCL1b *and *OsDCL1c *(Table 1, Figure 2C).

**Figure 3:**
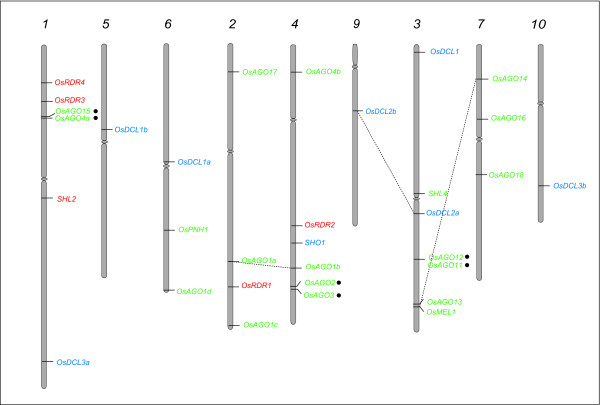
**Chromosomal localization of rice DCL, Argonaute and RDR genes**. Numbers on top designate the chromosome number for pseudomolecules. Segmentally duplicated genes have been joined using dashed lines, while tandem duplications are indicated by filled circles.

To gain insight into the evolution of multiple AGOs, RDRs and DCLs in rice, we analyzed their genomic distribution by localizing the genes on rice chromosomes (Figure 3; Table 1). Two pairs of rice Argonautes, *OsAGO14*-*OsMEL1 *and *OsAGO1a*-*OsAGO1b*, appear to have originated due to segmental duplications in chromosomes 3 and 7 and chromosomes 2 and 4, respectively. *OsAGO13 *was located close to *OsMEL1 *on chromosome 3, which has the maximum number of 5 genes, while the rest of the *OsAGO *were distributed on chromosomes 1, 2, 4, 5, 6 and 7 (Figure 3). Localization of *OsRDR *on rice chromosomes showed 3 genes, *OsRDR3*, *OsRDR4 *and *SHL2 *on chromosome 1 while chromosomes 2 and 4 had *OsRDR1 *and *OsRDR2*, respectively (Figure 3). None of these genes were found to be located in duplicated segments of the genome.

The *OsDCL *genes were distributed on 7 chromosomes (Figure 3). Except chromosome 3 that contains *OsDCL1 *and *OsDCL2a*, all the other chromosomes (1, 5, 6, 4, 9 and 10) have single representative of *OsDCL*. When these data were superimposed with those for segmental duplications (TIGR), *OsDCL2a *and *OsDCL2b *were found to lie in duplicated regions of chromosomes 3 and 9. It has been previously shown that at amino acid levels too, these two paralogs share 99% similarity while at the genomic level these genes differ by a 200 bp deletion within an intron and deletion of a part of Duf domain in OsDCL2b [10]. *OsDCL1a, OsDCL1b *and *OsDCL1c *that share significant homology were localized on regions of chromosomes 3, 5 and 6, which are not included in segmental duplication database, suggesting that events other than large segmental duplications might be responsible for their evolution. The expressed sequence tags (EST) showing similarity to these rice Dicer-like genes are present in *Zea mays *and *Saccharum officinarum *(AZM5_100042, CA252311) but not in *Arabidopsis*, thereby suggesting that duplication of *OsDCL1 *may have occurred after the divergence of monocots and dicots (~60 my).

A gene pair was considered tandemly duplicated if the members were separated by less than five intervening genes and shared ≥ 40% sequence similarity at amino acid level. Three *OsAGO *gene pairs, (*OsAGO4a-15; OsAGO2-3 and OsAGO11-12*) localized close to each other on chromosomes 1, 4 and 3, respectively, thus are considered to have arisen due to tandem duplications (Figure 3). They share 55%, 54% and 51% identity at amino acid level between the pair partners. None of the *OsDCLs *or *OsRDRs *seems to have undergone tandem duplications.

### Transcript profiling of genes during vegetative and reproductive development and abiotic stress

To analyze transcript abundance of Dicer-like, Argonaute and RDR genes at different stages of reproductive development in rice, microarray datasets generated in-house were utilized. The stages of vegetative and reproductive development analyzed in the present study are summarized [see Additional file 1]. Microarray hybridization was performed using the 57K Affymetrix GeneChip^® ^Rice Genome Arrays as described previously [27]. Normalization of data was done using GCRMA algorithm and was log_2 _transformed before differential expression analyses were undertaken. In the microarray dataset, > 99.9% of negative controls and non-rice probe-sets were found to have average signal intensity values less than 15. Therefore, the value '15' was considered as the cut off value to distinguish between expressed and non-expressed genes in a particular tissue/developmental stage.

**Figure 4:**
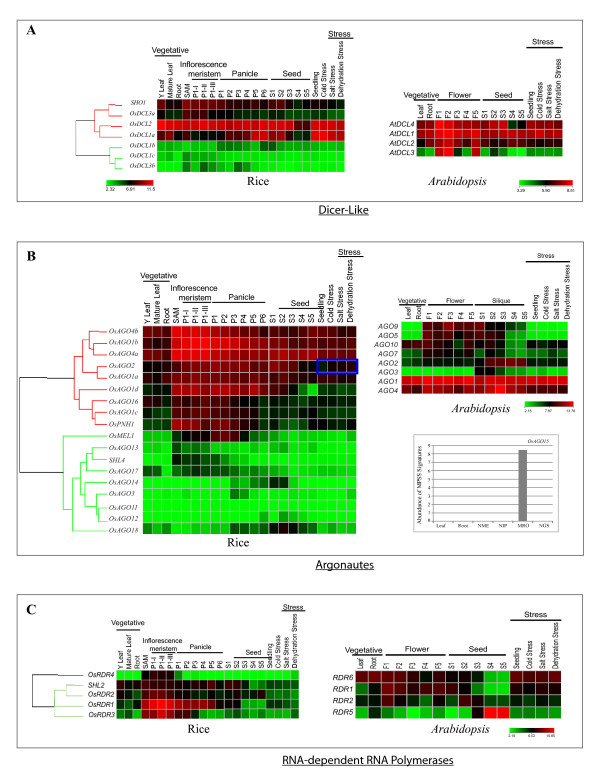
**Microarray based expression analysis of Dicer-like, Argonaute and RDR genes of rice and *Arabidopsis***. For rice genes the expression profiles have been analyzed in vegetative tissues (Y leaf, mature leaf, roots), Shoot Apical Meristem (SAM) and six stages of panicle development (P1 to P6) along with 3 substages of P1 i.e. P1-I, P1-II and P1-III, five stages of seed development (S1 to S5) and under three abiotic stress conditions (cold, salt and dehydration). For *Arabidopsis*, expression profiles of two vegetative stages (leaf and root), five stages of flower development (F1–F5), five stages of silique development and three stress treatments (cold, salt and dehydration) have been compiled. The color bar in each panel represents log_2 _expression values. Developmental stages used for expression profiling are mentioned on top of each column. Panicle and seed stages have been listed in the temporal order of development. On the left side of expression map, cluster dendrogram is shown. The bar graph in panel B shows the expression profiles of *OsAGO15 *based on MPSS data [41] from 60-days mature leaf, 60-days mature root, 60-days crown meristematic tissue (NME), 90-days immature panicle (NIP), mature reproductive organs (MRO) and 3-days germinating seed (NGS). Expression of *OsAGO2 *that shows two-fold up-regulation in response to all three stresses is boxed in blue in panel B.

The rice DCL genes *OsDCL1a*, *OsDCL2, OsDCL3a *and *SHO1 *were found to express ubiquitously (minimum normalized signal intensity values 67.7, 290.5, 32.0 and 82.2, respectively) in the tissues/stages of plant development analyzed [see Additional file 2]. These genes expressed at moderate to high levels in vegetative tissues but their expression was markedly reduced during specific stages of reproductive development (Figure 4A). Specifically, *SHO1, OsDCL1a *and *OsDCL2 *showed an approximately 10-, 37- and 10-fold down-regulation in late seed development stages (S4 and S5) in comparison to their peak expression in young seedlings, Y-leaf and SAM, respectively [see Additional file 3]. In contrast, two of the low expressing genes, *OsDCL1b *and *OsDCL3b*, exhibited panicle- and early seed-specific expression. The peak expression of *OsDCL1b *was found to be in P6 stage (corresponding to mature pollen stage of anther development) and was ~3-fold higher than its average signal intensity values in vegetative tissues. *OsDCL3b *expressed at ~6.5-fold higher levels in P1-II stage in comparison to its expression in vegetative stages. In vegetative tissues (except SAM), the average signal intensity values for both these genes were less than 15. Like *OsDCL1a*, *OsDCL2, OsDCL3a *and *SHO*, three *Arabidopsis *genes, *AtDCL1, AtDCL2 *and *AtDCL4*, were found to express in all the tissues/stages of development. On the other hand, *AtDCL3 *(like *OsDCL3b*) exhibited specific up-regulation of expression in reproductive stages, while in vegetative stages its expression was barely detectable. None of the *OsDCL *genes showed alteration in expression in response to the three abiotic stress conditions, viz. cold, salt or dehydration (Figure 4A).

**Figure 5:**
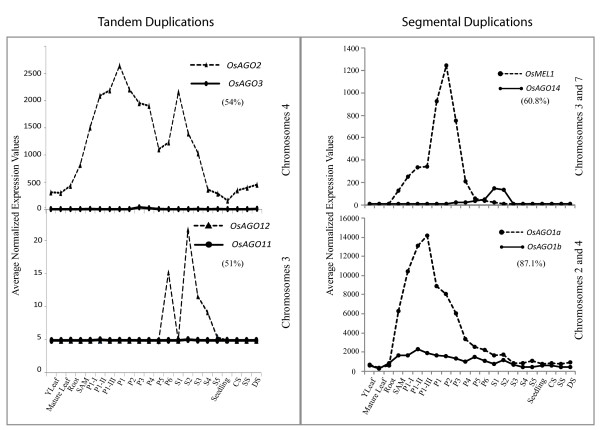
**Comparison of expression profiles of tandemly and segmentally duplicated Argonaute genes in rice**. Y-axis represents the average normalized expression values obtained from microarray analysis, while the X-axis depicts tissues/developmental stages [see Additional file 1]. Chromosomal location of each gene is mentioned on the right. Percentages in brackets indicate percentage of identical residues in the encoded proteins.

Among *OsAGOs*, 11 out of 19 genes (*OsAGO1a, 1b, 1c, 1d, 2, 4a, 4b, 13, 17, 18 and OsPNH1*) were found to express in both vegetative and reproductive tissues (Average signal intensities > 62.6; Figure 4B) [see Additional file 2]. Out of 19 genes, 14 genes belonging to *AGO1*, *AGO4 *and *MEL1 *clades (*OsAGO4b*, *1b*, *4a, 2, 1a, 1d, 16, 1c, OsPNH1, OsMEL1*, *13 *and *SHL4*), were significantly up-regulated (7-, 10-, 7-, 2.3-, 18-, 2-, 8-, 8-, 36-, 12-,11- and 36-fold, respectively) at the onset of floral development. Average signal intensity values of *MEL1 *clade genes, *OsAGO13 *and *OsMEL1*, were < 15 in vegetative tissues as compared to the other genes of *AGO1 *and *AGO4 *clades. *Arabidopsis AGO5 *has been shown to express only in reproductive tissues, i.e. during all stages of flower and seed formation [29]. This expression pattern is similar to that of *OsMEL1 *in rice. However, the expression of closely related *OsAGO13 *and *OsAGO14 *overlapped with that of *OsMEL1*. Individually, *OsAGO13 *expressed significantly in SAM and early stages of floral initiation (P1-I, P1-II and P1-III) and *OsAGO14 *expressed in late panicle and early seed development stages (Figure 4B). *OsMEL1 *and *OsAGO14 *share 60% identity at amino acid level and lie in duplicated segments of chromosomes 3 and 7 (Figure 3). However, their contrasting expression patterns during reproductive development suggest that these two genes have significantly diverged functionally after the duplication event (Figure 5). For the tandemly duplicated pair, *OsAGO11 *and *OsAGO12*, in this clade, only *OsAGO12 *transcripts could be detected during late panicle (P6) and early seed stages (S1–S4) while transcripts for *OsAGO11 *were barely detectable at any of the stages (Figure 5). In *AGO1 *subgroup, there are 5 rice genes clustered with two *Arabidopsis *genes, *AGO1 *and *AGO10*. Unlike their rice counterparts, *Arabidopsis AGO1 *and *AGO10 *expressed in vegetative as well as reproductive tissues without any significant increase in transcript accumulation in reproductive tissues. Whereas, all the rice genes in this subgroup exhibited 3- to 36-fold enhancement in transcript levels coinciding with the initiation of reproductive development. Expression pattern of the segmentally duplicated genes, *OsAGO1a *and *OsAGO1b*, in this clade overlapped at all stages and in all tissues, with *OsAGO1b *being expressed at relatively higher levels than *OsAGO1a *(Figures 4B and 5). In the ZIPPY clade, expression of tandemly duplicated genes, *OsAGO2 *and *OsAGO3*, overlapped only during P3–P5 stage of panicle development while at all the other stages, only *OsAGO2 *was observed to be highly expressed while, *OsAGO3 *transcripts could not be detected (Figure 4 and 5). This shows that both segmental and tandem duplication of Argonaute genes have resulted in formation of gene partners that have probably diverged functionally during the course of evolution and have thus contributed to the diversity of this protein family in rice. Members of ZIPPY subgroup, *SHL4/SHO2 *and *AGO7 *differed from other members of this clade in being expressed predominantly during early stage of floral development. There are four genes each from rice and *Arabidopsis *that constitute the *AGO4 *clade. Out of these, the coding region of *OsAGO15 *is interrupted by a retrotransposon. Amongst the *Arabidopsis *genes, expression profiles of only *AGO4 *and *AGO9 *could be obtained. On the basis of transcript accumulation patterns and sequence similarities, the *Arabidopsis AGO4 *seems to have two counterparts in rice, *OsAGO4a *and *OsAGO4b*. These three genes showed enhanced expression during early stages of floral development. The *OsAGO4a *and *OsAGO4b *were up-regulated by 6.9 and 6.7 folds, while the enhancement was only 2.6 folds in case of *AtAGO4 *(Figure 4B) [see Additional files 3 and 4]. Conservation in expression profiles was also observed for *Arabidopsis AGO2 *and *OsAGO2 *genes, whereas, the expression of *AGO3 *gene differed in both rice and *Arabidopsis. AtAGO3 *expressed specifically during stages of seed development, while expression of *OsAGO3 *was confined to later stages of panicle formation (P3–P5). *OsAGO18*, that did not cluster into any of the four subgroups exhibited high level expression during late panicle (P3–P6) and seed development stages, S1–S5, along with low level expression in vegetative tissues. For *OsAGO11*, a unique Affymetrix probe set ID existed, but no transcripts could be detected in any of the tissues or developmental stages analyzed. Most *OsAGOs *did not show any variation in expression in response to the three abiotic stresses, except *OsAGO2*, whose transcript levels increased by > 2-folds (p-value ≤ 0.05) in response to all three stresses.

Of the five *OsRDR *genes, four expressed both in vegetative and floral tissues with average intensity values of > 15 in any or all stages of vegetative, floral- and seed-development (Figure 4C). However, a significant increase in the transcript abundance for *OsRDR1*, *OsRDR2 *and *OsRDR3 *(56-, 4.2- and 26.6-fold, respectively, at p-value ≤ 0.05) was observed during stages of early panicle development (P1-I to P1-III) in comparison to their expression in mature leaves (Figure 4C) [see Additional files 3 and 4]. The expression of *OsRDR1 *was detectable up to S2 stage of seed formation and declined thereafter. The expression profile of the closely related *Arabidopsis *genes, *RDR1 *and *RDR2*, did not show significant increase in transcript levels as compared to rice genes during early flower development; nevertheless, their transcripts were detectable until early stages of seed development. Unlike any other corresponding gene in *Arabidopsis*, the expression of *OsRDR4 *(that did not show any detectable expression in vegetative tissues except SAM) was found to be up-regulated by 36 folds in early stages of panicle development in comparison to mature vegetative tissues.

**Figure 6:**
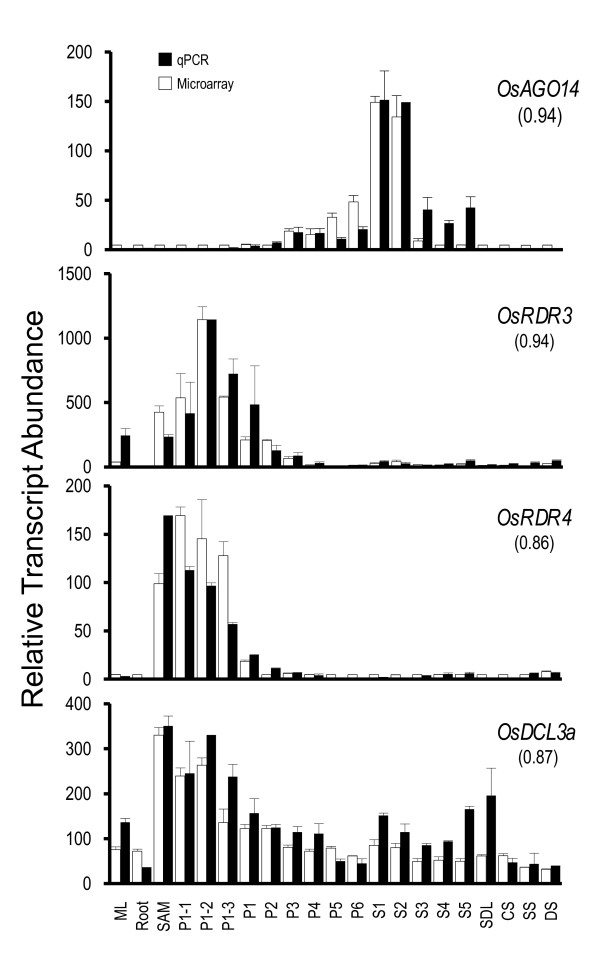
**QPCR results for selected four genes and its correlation with microarray data**. Two and three biological replicates have been taken for QPCR and microarray, respectively. Standard error bars have been shown for data obtained using both the techniques. Y-axis represents raw expression values obtained from microarray analysis, QPCR data has been normalized to ease profile matching with that of microarrays. X-axis depicts tissues/developmental stages as described in Figure 4 [see Additional file 1]. For each gene the Pearson's correlation values between the two analyses are shown in parenthesis.

Four genes, *OsAGO14, OsRDR3, OsRDR4 and OsDCL3a*, showing discrete expression patterns were selected for validation of microarray expression profiles by QPCR analysis. Figure 6 shows a comparison of the QPCR and microarray analysis. The expression patterns obtained for all four genes using QPCR were similar to that derived from the microarrays with Pearson's correlation values ranging from 0.86 to 0.94 (Figure 6).

## Discussion

RNA interference plays an important role in regulating gene expression at post transcriptional level during vegetative and reproductive development in plants [6]. This process is mediated by concerted activities of proteins like, Dicers, Argonautes and RNA-dependent RNA polymerases. In the present investigation, we have identified the genes encoding these proteins families in rice and studied their distribution on rice chromosome pseudomolecules. Phylogenetic analysis has shed light on the evolution of members in each gene family in rice and has provided insight into multiplicity of Argonaute genes in rice. Microarray based expression analysis at different stages of reproductive development in rice plants was attempted with the view to understand the contribution and developmental timings of activity of these proteins.

### Rice Dicer-like genes

While there is only one Dicer in mammals and nematodes that is involved in processing all sizes of small RNAs, four *DCL *genes found in *Arabidopsis *seem to have specialized in the type of small RNAs they process [10]. For example, *DCL1 *and *DCL4 *are required for biogenesis of 21 nt small RNAs that correspond mainly to miRNA and tasiRNA, *DCL2 *affects accumulation of 21 nt RNA that protects against viral infection and *DCL3 *acts to produce the 24 nt RNAs that mediate *de novo *DNA methylation, gene silencing and chromatin modification [19,30]. We have identified all four classes of *DCL *genes in rice, that total eight in number. While duplication of *DCL2 *genes (*OsDCL2a *and *OsDCL2b*), which share more than 90% similarity at the amino acid level, has also been reported in *Arabidopsis *and *Populus trichocarpa*. However, duplication of *DCL3 *that gave rise to *OsDCL3a *and *OsDCL3b *is considered specific to monocots and predates the divergence of rice and maize [10]. In the present study, microarray based expression pattern analysis of rice and *Arabidopsis DCL *genes at different stages of reproductive development shows conservation in expression pattern of genes in dicot and monocot plants, however, duplication events in rice may also have given rise to genes with novel expression (and probably functional) profiles.

Null mutants of *AtDCL1 *exhibit pleiotropic developmental defects and are lethal due to loss of accumulation of miRNAs. Henderson et al. (2006) also demonstrated that miRNAs dominate the pool of small RNAs (67.5%) accumulated in *dcl2*/*dcl3*/*dcl4 *triple mutants [19]. In rice too, it has been shown that loss-of-function of *OsDCL1 *but not *OsDCL4 *affects processing of miRNA [25]. Consistent with the roles of miRNAs in regulation of various plant developmental processes, *AtDCL1 *was found to express ubiquitously in all tissues. The presence of three *DCL1 *like genes in rice with divergent expression profiles is suggestive of diversification of DCL1 function in rice.

*AtDCL3 *together with *RDR2 *in *Arabidopsis *is known to affect accumulation of small RNAs that mediate *de novo *DNA methylation and transgene silencing. In rice, of the two OsDCL3s, OsDCL3a was previously considered as the functional counterpart of AtDCL3 on the basis of overall homology and conservation of dsRBb domain. On the other hand, the dsRBb domain in OsDCL3b was so divergent from that of AtDCL3 that it was suggested to categorize it as an altogether different, fifth class of dicer-like protein [10]. *AtDCL3 *and *OsDCL3b *show low level expression in vegetative tissues with enhanced transcript accumulation in floral tissues and early stages of seed development. However, *OsDCL3b *expression was confined only to early (pre-meiotic) stages of panicle development. Hence, on the basis of expression profiles *AtDCL3 *and *OsDCL3b *share similar expression domains, although the encoded proteins possess highly divergent dsRBb domains and could therefore be interacting with different RNA substrates.

*Arabidopsis DCL4 *has been shown to function redundantly with *DCL2 *and *DCL3 *to process *SHL2(OsRDR6) *generated double stranded RNA into tasiRNA The RNA template for *SHL2 *is in turn produced by the activity of miR173 and miR390 generated by *DCL1*, *HYL1*, *HEN1 *and *AGO1 *[30]. The expression patterns of rice genes, *OsDCL4*, *SHL2 *and *OsAGO1 *members (*OsAGO1a*, *OsAGO1b*, *OsAGO1c *and *OsAGO1d*) matched that of the corresponding genes in *Arabidopsis *especially in exhibiting enhanced expression during early stages of floral/panicle development. This further suggests that the diversification in function, as speculated from their expression pattern, of most plant *DCL *genes may have occurred after the split of plants and animal lineages but before the divergence of monocots and dicots.

### Multiple AGOs and their roles in rice plant development

Argonautes form an evolutionarily conserved gene family whose members are present in both single celled and multicellular eukaryotes. Multiple genes have been reported in various organisms. Rice has the largest number of Argonautes among plants, almost double the number reported in *Arabidopsis *and second only to *C. elegans *that possesses 27 AGOs [14]. Rice genes with similar/overlapping expression domains with *Arabidopsis AGO1*, (*OsAGO1a*, *OsAGO1b*, *OsAGO1c *and *OsAGO1d) AGO5*, (*OsAGO13*, *OsAGO14 *and *OsMEL*), and *AGO4*, (*OsAGO4a *and *OsAGO4b*), have been identified in the present study. Most of these genes appear to have evolved by duplication events (tandem or segmental) followed by differentiation of expression patterns. These duplication events may also have given rise to redundancy among *OsAGO *genes as is evident from the overlapping expression profiles of *OsAGO1a*, *OsAGO1b*, *OsAGO1c *and *OsAGO1d *and *OsAGO4a *and *OsAGO4b *genes. On the other hand, *OsMEL1 *and *OsAGO14*, that appear to have arisen by duplication of segments in chromosomes 3 and 7, exhibit contrasting expression profiles during panicle and seed development.

Argonautes are highly basic RNA binding proteins characterized by presence of PAZ and PIWI domains. The PIWI domain adopts an RNAse-like fold and has predicted endonuclease activity (slicer). It binds to 5' end and also interacts with the target RNA [31,32]. The PAZ domain interacts with 3' of small RNAs and is the candidate region that determines the specificity of Argonautes. Consistent with this, homology among various rice AGOs was observed to be minimum in the PAZ regions (~21%) while their PIWI domains exhibited more than 90% conservation of amino acids. The endonuclease property of AGO proteins involved in RNAi resides in the PIWI domain that possesses three conserved metal-chelating amino acids (DDH). Subfamilies of AGO proteins, primary and secondary Argonautes, have been described in *C. elegans *on the basis of presence or absence of these catalytic amino acid residues [14]. Five of the *Arabidopsis *AGO proteins, AGO2, AGO3, AGO4, AGO6 and AGO9 lack either the critical DDH or the H798 residue. In rice too, eleven genes do not code for the conserved catalytic residues in their PIWI domains. Of these, *OsAGO3*, *OsAGO5a*, *OsAGO5b *and *OsAGO15*, are expressed specifically or preferentially in reproductive tissues. Absence of catalytic amino acids could inhibit the processing of target RNA by endonucleolytic cleavage in these proteins. These proteins could, therefore, require accessory factors for mediating mRNA turnover. However, it has been reported that an aspartate at the third position of the catalytic triad in related RNase proteins, Integrase and Tn5, can chelate the divalent metal ions as efficiently as histidine residue and restore the catalytic activity [32,33]. Accordingly, *AGO2 *and *AGO3 *of rice and *Arabidopsis *that possess DDD could still function as slicer components of silencing effector complexes. In rice, while *OsAGO2 *is ubiquitously expressed, *OsAGO3 *along with *OsAGO14*, *OsAGO13 *and *OsMEL1 *are expressed specifically in reproductive tissues. These genes can, therefore, be considered as candidate genes regulating expression of endogenous genes via RNA interference that may or may not involve cleavage of target RNA as their mode of activity.

In rice, knockout studies by three groups have provided insights into the functions of three *OsAGO *genes, *OsMEL1*, *SHL4/SHO2 *and *OsPNH1 *[15,26,34]. OsMEL1 is a unique AGO protein that regulates meiosis in germ cells and proper development of male and female gametes by chromatin modification probably mediated by H3K9 methylation. Whether OsMEL1 directly interacts with regulatory small RNAs to bring about desired changes at the chromatin is not yet known. *OsMEL1 *also has a duplicated partner, *OsAGO14*, located on chromosome 7 that does not show any expression in vegetative tissues and is specifically expressed at stages beginning from P3 in panicles and until S2 stage in seeds. Furthermore, diversity in their PAZ domains suggests that these two proteins could interact with different classes of small RNAs, while retaining the mode of processing the target RNA by virtue of the catalytic DDH motif in their PIWI domains.

The rice *SHL4*, *SHL2 *and *SHO1 *genes have been shown to be involved in initiation and formation of SAM during rice embryogenesis. This is mediated by miRNA and tasiRNA regulation of homeodomain-leucine zipper (HD-ZIPIII) and ETT/ARF gene families [26]. *OsPNH1*, is the third gene whose functional analysis revealed that it affects the development of SAM and leaf development [34]. This gene is closely related to *OsAGO1*.

### Rice RDR genes in panicle development

RNA-dependent RNA polymerases are the regulatory components of RNAi machinery, which enhance the potency of RNAi by amplifying the aberrant RNA population [35]. These proteins are required for both cytoplasmic gene silencing that is triggered by transgenes or viral infection, as well as, nuclear gene silencing for transposons and inverted repeats in the genome. In *Arabidopsis*, 6 genes encode RDRs out of which only 3 genes have been shown to be involved in viral defense, chromatin silencing and PTGS. *RDR6*, also known as *SGS2 *and *SDE1 *amplifies improper terminated and unpolyadenylated RNAs generated from transgenes or inverted repeats to trigger degradation of complementary RNA species [36]. Genetic studies in *Arabidopsis *have also revealed that *RDR6 *functions in the same pathway as *AGO7 *and *Asymmetric leaves 1 *and *2 *(*AS1 *and *AS2*) to control adaxial/abaxial patterning in leaves [37]. This role of *RDR6 *appears to be evolutionarily conserved in dicot and monocot lineages as in rice it was recently reported that *SHL2 *along with *SHL4*, and *SHO1 *affect siRNA and tasiRNA mediated regulation of endogenous genes involved in SAM and leaf development [26]. *Arabidopsis RDR2 *is required for combating viral infection through PTGS and is involved in siRNA mediated de novo methylation of direct repeats. Recently, it has been shown that the homolog of *Arabidopsis RDR2 *in maize, *MOP1*, is involved in paramutation of b1 locus [38]. The *MOP1 *gene maintains a threshold level of RNA encoded by repeat sequences that modify chromatin at b1 locus. This gene is also closely related to *OsRDR2*. The role of *OsRDR2 *is not known yet, however its expression was observed to overlap with *Arabidopsis RDR2 *at earlier stages of flower development and it phylogenetically relates to both maize and *Arabidopsis *genes. It will therefore be interesting to study the role of this gene in rice to validate the speculation of conservation of their roles, based on their similar expression patterns, in dicots and monocots.

## Conclusion

Regulation of gene expression at post-transcriptional level plays a critical role in plant development. RNA interference mediated by activities of Dicers, Argonautes and RNA-dependent RNA polymerases is an important regulatory process that checks transcript accumulation in cells. These components are also shared by other gene-silencing pathways. Dicer-like, Argonautes and RDRs are encoded by small multigene families and rice encodes the largest number of these genes among the plant species analyzed so far. Phylogenetic analysis and localization on rice pseudomolecules have revealed that duplication of genes, both segmental and tandem, have contributed to increase in number of these genes in rice. However, function of only a few of these genes has been established in both rice and *Arabidopsis*. A key objective of this study was to generate and compile an expression profile data set to facilitate selection of candidate genes for validation of their roles during reproductive phase of plant development and abiotic stress responses. More than 20 genes belonging to these three gene families were observed to express preferentially/specifically during panicle and seed development. This work has provided insights into the probable domains of activity of these genes.

## Methods

### Identification of genes, chromosomal localization and phylogenetic analysis

Name search and Hidden Markov Model (HMM) analysis was employed to search for Dicer-like, Argonautes and RDRs genes encoded in the rice genome. The sequences were downloaded from TIGR, release 5 . An HMM profile was generated using HMMER 2.1.1 software package  which was then used to search the proteome database of rice available at TIGR using the Basic Local Alignment Search Tool, BLAST with filter off. Amino acid sequences of all the rice genes were downloaded and conserved domains were searched using the Simple Modular Architecture Research Tool (SMART) version 3.4 or National Center for Biotechnology Information Conserved Domain Database, NCBI-CD [39]. The newly identified genes in this study were named on lines of nomenclature used for the previously identified genes and on the basis of their phylogenetic relatedness to other members of the same family. ORF length, and details of encoded proteins (length, PI, molecular weight) were downloaded from TIGR. For proteins whose molecular weight and PI was not available in TIGR, Gene Runner program version 3.04 was used to calculate the same. Genes were localized on the chromosomes based on their chromosomal positions on psedudomolecules as mentioned in TIGR. For phylogenetic analysis, total protein sequences were downloaded from TIGR and aligned using ClustalX 1.83 program. An unrooted neighbor-joining tree was constructed in ClustalX using default parameters [40]. Bootstrap analysis was performed using 1000 replicates.

### Plant material

Plant tissue for all the panicle and seed stages as well as mature leaf and Y-leaf was collected from field grown rice plants (*Oryza sativa ssp. indica *var. IR64). Rice seedlings were subjected to abiotic stress treatments, viz. salt, drought and cold as described previously [27].

### Microarray Hybridization and Data Analysis

Affymetrix GeneChip^® ^Rice Genome Arrays representing 49,824 rice transcripts were used to prepare a compendium of transcriptome profiles for 22 stages of vegetative and reproductive development and stress response in rice. Of these, microarray analysis of 17 stages was described previously [27]; deposited in the Gene Expression Omnibus database at the National Center for Biotechnology Information under the series accession numbers GSE6893 and GSE6901). Here, five more stages viz, Y-leaf, SAM (shoot apical meristem), P1-I (< 2 mm panicle), P1-II (0.2 to 0.5 mm panicle) and P1-III (5 to 10 mm panicle) have been added to emphasize changes in gene expression patterns during initial stages of panicle differentiation and floral primodia emergence in addition to stages of floral organ- and seed development. Total RNA from these five stages was isolated by using TRIzol method (Invitrogen Inc., USA; [18]). After checking the quality on agarose formaldehyde gels, the RNA samples were quantified using spectrophotometer (ND-1000, Nanodrop). Five micrograms of RNA with 260:280 ratios of 1.9–2.0 and 260:230 ratios more than 2.0 was used for cDNA synthesis. Labeling and hybridizations were carried out according to Affymetrix manual for one-cycle target labeling (Affymetrix, Santa Clara, CA). Hybridization was performed in GeneChip^® ^Hybridization Oven 640 for 16 hours at 45°C and 60 rpm. GeneChips were washed and stained with streptavidin-phycoerythrin using the fluidics protocol EukGE_WS2V5_450 in Affymetrix fluidic station model 450. Finally, chips were scanned using the Gene-Chip^® ^Scanner 3000-6G.

Sixty six cell intensity (CEL) files generated by GeneChip Operating Software (GCOS) were further analyzed using Arrayassist™ version 5.0 (Stratagene, La Jolla). Data were normalized using GC-RMA algorithm and log_2 _transformed. To get the expression values, averages of three biological replicates were used. The expression data for Dicer-like, Argonaute and RDR genes was extracted by using unique probe-set IDs mentioned for each gene in TIGR. Wherever more than one probe set was available for one gene, the probe set designed from 3' end was given preference. Cluster analysis on rows of expression values was performed by using Euclidean distance metric, and Ward's Linkage rule of hierarchical clustering. Differential expression analysis was performed by taking mature leaf as reference to identify genes expressing at more than two-fold level in different stages of reproductive development (panicle and seed), with p-values ≤ 0.05. Similarly, for identifying stress-induced genes, differential expression analysis was performed by taking seedling as reference with no correction applied and p-values less ≤ 0.05 [see Additional file 3]. Since the Affymetrix microarrays used in this study did not contain probes representing *OsAGO15*, data for this gene was extracted from rice MPSS database .

### Arabidopsis expression analysis

To analyze the expression of *Arabidopsis *genes, Affymetrix GeneChip^® ^ATH1 Genome Array data for 21 stages (55 .cel files) comparable to that used for rice were downloaded from Gene Expression Omnibus (GEO) database at the NCBI under the series accession numbers GSE5620, GSE5621, GSE5623, GSE5624, GSE5629, GSE5630, GSE5631, GSE5632 and GSE5634. The data were imported in ArrayAssist™ (Stratagene, La Jolla, CA) microarray analysis software wherein GCRMA algorithm was used for normalization and Log_2_transformation. [see Additional file 4]. The downstream processing and generation of heat maps for selected genes was as described for rice.

### QPCR analysis

Real time PCR reactions were carried out by using the same RNA samples, which were used for microarrays as described earlier [27]. In brief, primers were designed preferentially from 3' end of the genes by using PRIMER EXPRESS version 2.0 (PE Applied Biosystems, USA) with default parameters. Each primer was checked by using BLAST tool for homology with other regions of the genome. 4 μg of total RNA was used for first strand cDNA synthesis in 100 μl reaction volume by using high-capacity cDNA Archive kit (Applied Biosystems, USA). Diluted cDNA samples were used for Real time PCR analysis with 200 nM of each primer mixed with SYBR Green PCR master by using ABI Prism 7000 Sequence Detection System and software (PE Applied Biosystems, USA). Actin was used as endogenous control to normalize the variance among samples. Relative expression values were calculated after normalizing against the maximum expression value. These data were further normalized with the normalized expression values obtained from microarrays and bar charts plotted by using Microsoft Excel.

## Authors' contributions

RA and AN generated expression data for mature leaf, panicle and P1-I, P1-II and P1-III stages under the supervision of JPK and AKT. AKT also extended the use of microarray facility. TL participated in computational analysis of gene families, MK and SK conceptualized the analyses, performed computational analysis; MK drafted the manuscript and SK participated in and supervised all the experiments related to panicle development and revised the final version of the manuscript. All authors read and approved the final manuscript.

## Supplementary Material

Additional file 1 Developmental stages/organs of rice plant analyzed. Click here for file

Additional file 2 GCRMA normalized expression values obtained for Dicer-like, Argonaute and RDR genes by using rice microarrays data. Click here for file

Additional file 3 Differential expression analysis of rice genes in 19 stages/tissues of vegetative and reproductive development by taking mature leaf, root, seedling, SAM and Y-leaf individually as reference. Click here for file

Additional file 4 GCRMA normalized expression values for Dicer-like, Argonaute and RDR genes in *Arabidopsis *from microarray data in public domain (Gene Expression Omnibus, ). Click here for file
